# Placement of Triangular Titanium Implants Suprajacent to Pelvic Fixation Screws Reduces Pelvic Fixation Failure in Multilevel Spinal Fusion: Results of a Randomized Controlled Trial

**DOI:** 10.2106/JBJS.25.01415

**Published:** 2026-07-16

**Authors:** Benjamin D. Elder, Gregory Mundis, Robert Eastlack, Paolo Arrigoni, Joerg Franke, Robert Lee, David Polly

**Affiliations:** 1 Mayo Clinic, Rochester, Minnesota; 2 San Diego Spine Foundation, San Diego, California; 3 ASST-Ovest Milanese, Legnano, Lombardy, Italy; 4 Klinikum Magdeburg, Magdeburg, Germany; 5 Royal National Orthopaedic Hospital, Stanmore, United Kingdom; 6 University of Minnesota, Minneapolis, Minnesota

## Abstract

**Background:**

The purpose of this study was to determine whether placement of triangular titanium implants (TTI) suprajacent to S2-alar-iliac (S2AI) pelvic fixation screws during multilevel spinal fusion surgery for adult spine deformity reduces pelvic fixation failures.

**Methods:**

This prospective, multicenter, international, partially blinded randomized clinical trial included 222 patients scheduled for multilevel spinal fusion surgery with S2AI-based pelvic fixation. In the S2AI+TTI group, the mean age was 64.9 years, 54.1% were female, 3.7% were Hispanic, 5.5% were African American, and 1.8% were Asian. In the S2AI-alone group, the mean age was 64.8 years, 58.4% were female, 3.5% were Hispanic, 7.1% were African American, and 0.9% were Asian. Subjects were randomized to pelvic fixation using S2AI screws alone versus S2AI+TTI. Scheduled follow-up included a 24-month full-spine radiograph and a high-resolution computed tomography (CT) scan, which was independently interpreted.

**Results:**

The primary composite radiographic end point (S2AI screw breakage, lucency, or pullout, or posterior spinal rod breakage distal to S1) occurred less frequently in the S2AI+TTI group than in the S2AI-alone group (11.0% versus 20.4%, p = 0.0415). Except for rod-screw dissociation and rod fracture distal to S1, all components of the primary composite end point occurred less frequently in the S2AI+TTI group. TTI placement did not affect sagittal or coronal spinal alignment parameters. The rate of rod fracture throughout the spinal construct was slightly higher in the S2AI+TTI group (9.2% versus 5.3%) but the difference was not significant (p = 0.197).

**Conclusions:**

Placement of TTI suprajacent to S2AI screws reduced the rate of pelvic fixation failure in patients undergoing multilevel spinal fusion with pelvic fixation.

**Level of Evidence:**

Therapeutic Level I. See Instructions for Authors for a complete description of levels of evidence.

Adult spinal deformity (ASD) occurs commonly in patients ≥65 years old^1^ and greatly impacts quality of life^2^. Complex spinal fusion procedures are the primary means for addressing severe deformity^3-5^.

In patients undergoing fusion of >4 spinal segments, pelvic fixation is commonly used to improve fusion rates at the L5/S1 level, reduce instrumentation failure, and maintain post-correction alignment. Methods for pelvic fixation have evolved substantially over the past decade^6,7^. Current methods include placement of ≥1 screws directly into the ilium or through the sacroiliac joint (SIJ)─i.e., the sacral-alar-iliac (SAI) trajectory.

Pelvic fixation failures are common. Acute (<90 days) failures include set plug dissociation, tulip head dissociation, pelvic screw fracture, rod fracture, distal fracture of the sacrum or pelvis, and offset connector failure; these occur in approximately 5% of cases^8,9^. Delayed complications are also common^10-14^, with long-term rates ranging from 34% to 47%^15,16^. Revision surgery is expensive^17^. Thus, construct strategies that reduce pelvic fixation failures may benefit patients and be cost-effective.

While placement of a single pelvic fixation screw is common, placing 2 devices in the SAI trajectory can reduce SIJ motion^18-20^ and reduce stress on the instrumentation^18^, and in 1 study it reduced the screw failure rate from 9% to 3%^21^. Placement of triangular titanium implants (TTI) suprajacent to pelvic fixation screws was introduced in 2019^22^. TTI stabilize the SIJ via multiple points of fixation^19,23^, integration into host bone^24^, and SIJ fusion^24^. To confirm the benefits of this, we conducted a comparison of pelvic fixation using S2AI screws (SAI screws placed through S2) alone versus S2AI screws with concomitant suprajacent TTI placement (S2AI+TTI) (Fig. 1). In a companion clinical study, we observed a lower rate of postoperative SIJ pain (the study’s primary clinical end point) in the S2AI+TTI group (1.1% versus 8.5%)^26^. Herein, we report a comparison of radiographic failures.

**Fig. 1:**
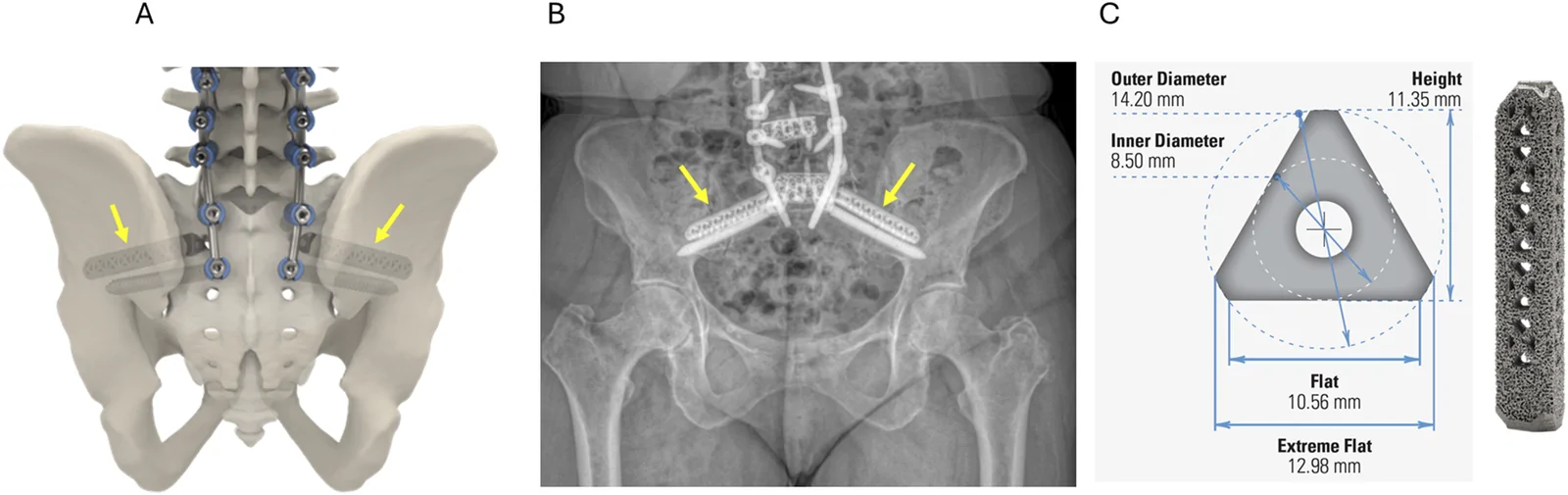
**Figs. 1-A and 1-B:** Triangular titanium implants (TTI) placed suprajacent (yellow arrows) to S2AI pelvic fixation screws. **Fig. 1-C:** TTI detail and dimensions.

## Materials and Methods

SILVIA (ClinicalTrials.gov NCT04062630) is a prospective, multicenter, international, partially blinded randomized controlled trial conducted at 29 sites in the U.S., Europe, and Australia. Approvals from local ethics boards were obtained at each site prior to the study start, and all subjects provided written informed consent. Study data were monitored and source data were verified by the study sponsor, SI-BONE, Inc., which is the manufacturer of the iFuse 3D implant, the TTI used in the study.

### Screening and Baseline

Patients aged 21 to 75 years with spinal deformity who were scheduled for multilevel spinal fusion (≥4 levels) with planned S2AI pelvic fixation were included in the study (Fig. 2). Patients were excluded for the following reasons: congenital spinal deformity as an indication for surgery, prior pelvic fixation, grade-IV or greater spondylolisthesis, sacral or iliac pathology (e.g., tumor) or anatomy that could prevent placement of S2AI screws and/or triangular implants, severe osteoporosis (e.g., DXA [dual x-ray absorptiometry] T-score below −3.5), allergy to titanium, presence of an active spinal cord stimulator, hip pain suggesting severe hip osteoarthritis, use of medications that impair bone healing, a neurologic deficit that would interfere with postoperative recovery, a Workers’ Compensation claim related to SIJ or back pain, a severe psychiatric condition, or fibromyalgia. Clinical outcomes have been reported on previously^26^.

**Fig. 2:**
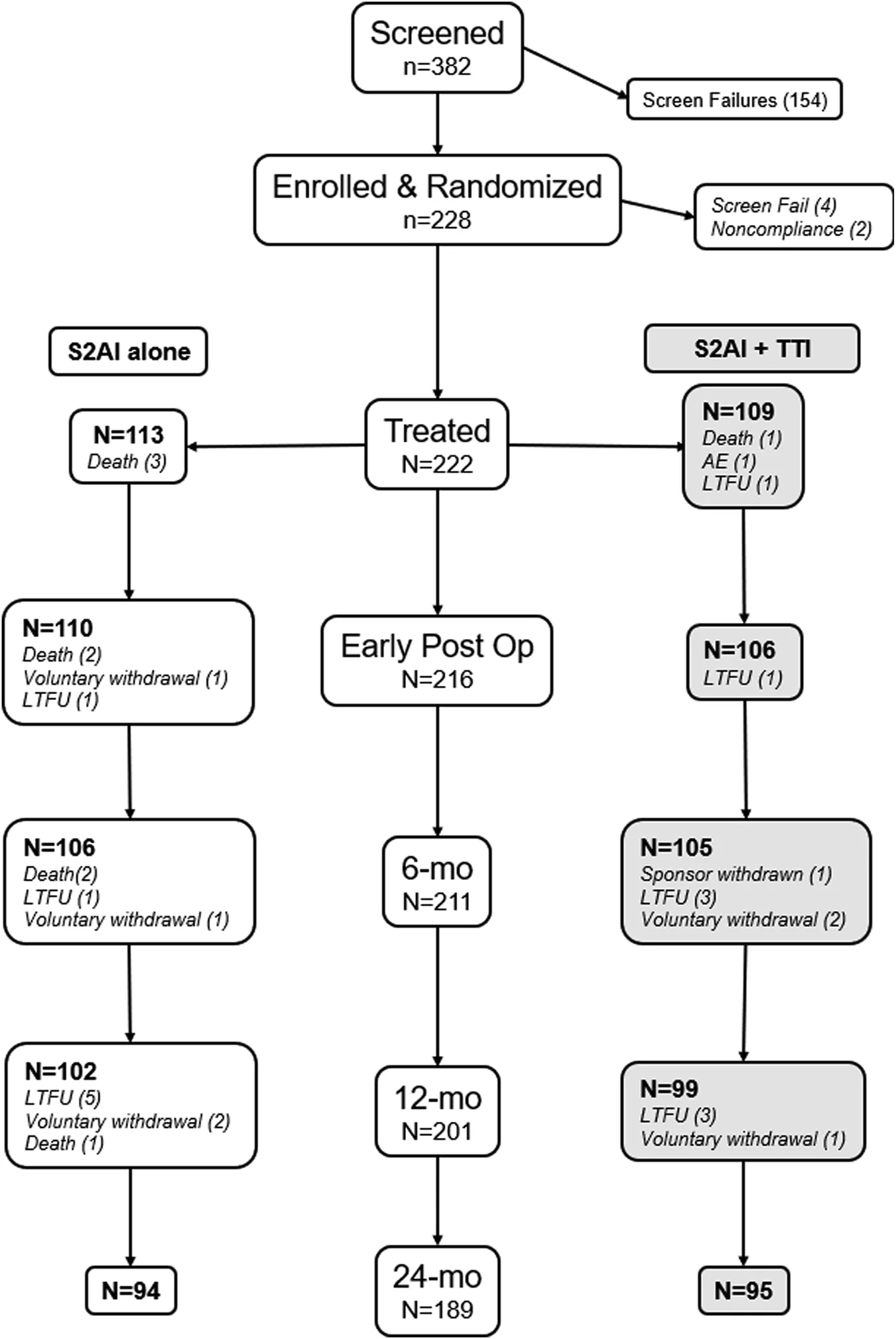
CONSORT diagram. LTFU = loss to follow-up, AE = adverse event.

### Randomization

Subjects were randomized 1:1 to S2AI alone or S2AI+TTI, with the TTI placed suprajacent to each screw^25^. A randomization minimization algorithm used stratifications by the planned upper instrumented vertebral body (UIV) and presence or absence of preoperative SIJ pain^26^.

### Follow-up

Subjects were evaluated at 6, 12, and 24 months postoperatively. At month 24, a standing full-length spinal radiograph and high-resolution computed tomography (CT) scan of the pelvis were acquired. Imaging of any observed instrumentation failures was also reviewed.

### Independent Radiographic Review

An independent radiologist adjudicated the following: (1) for S2AI and S1 screws: breach, lucency, tulip failure, set screw dissociation; (2) any posterior spinal rod breakage; (3) for TTI: whether TTI were placed at least 20 mm into the ilium, whether the distal end of the TTI was outside of the ilium, osseous apposition to the TTI in the ilium and sacrum; (4) for the SIJ: presence or absence of bridging bone across the SIJ; and (5) for all instrumentation: any adverse bone reactions. Surgimap (Globus) was used to assess sagittal and coronal spinal alignment parameters.

All observed pelvic instrumentation lucencies were additionally scored using a modified Shimizu-Lenke^27^ classification system (Fig. 3). The grader rated lucency at both the sacral and iliac components of each implant. There was nearly complete agreement on lucencies between the independent reviewer and the senior author (D.P.).

**Fig. 3:**
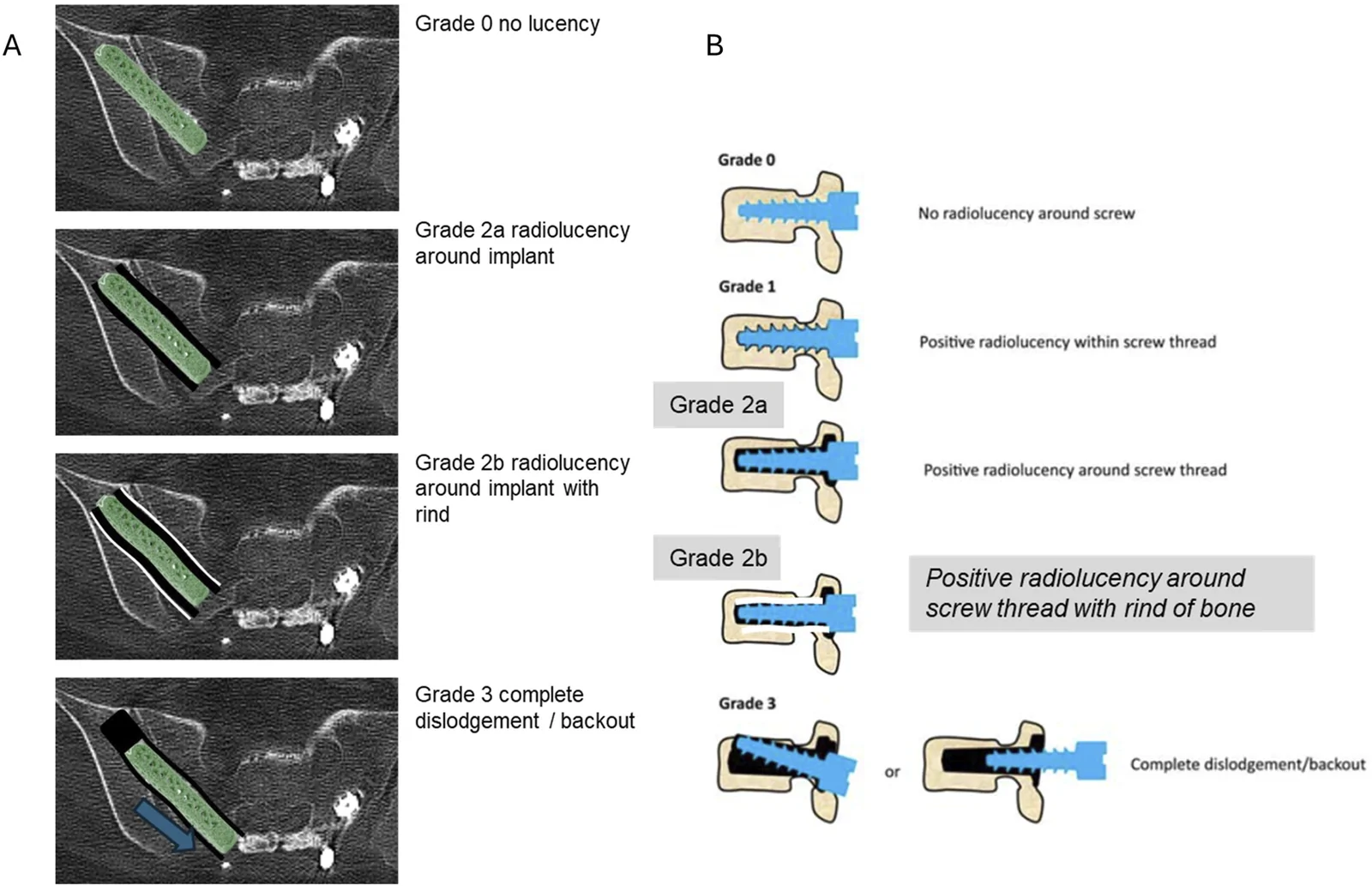
**Fig. 3-A:** Modified Shimizu-Lenke system as applied to TTI. Because TTI do not have threads, there is no Grade 1. **Fig. 3-B:** Modified Shimizu-Lenke^27^ system for screw lucency as applied to S2AI implants, in which Shimizu-Lenke Grade 2 was called 2a and any screw showing both lucency around the screw thread and a surrounding rind of bone was called 2b.

### End Points

The primary radiographic end point, which reflects pelvic fixation failures, was a composite of any of the following components: (1) S2AI screw breakage, lucency, or pullout, or (2) rod breakage distal to S1. An additional analysis of the primary radiographic end point was performed in which its first component was expanded to additionally include S1 screw breakage, lucency, or pullout.

### Sample Size

A preplanned sample size of 100 subjects per group was calculated to result in 80% power to detect a 50% relative reduction in the primary radiographic end point from 30% to 15%. The overall sample size was increased to 220 to account for 10% potential loss to follow-up at 2 years.

### Statistical Analysis

All analyses were conducted in R (version 4.2.1; The R Foundation)^28^. Binary outcomes were compared using the Fisher test or Cochran-Armitage trend test^29^. Continuous variables were compared using t tests. All statistical hypothesis testing for the end point analyses was 1-sided (i.e., testing whether rates of undesired radiographic outcomes in the S2AI+TTI group were lower than those in the S2AI-alone group).

## Results

Of the 222 subjects enrolled and treated between May 2020 and October 2022, 113 were assigned to the S2AI-alone group and 109 were assigned to the S2AI+TTI group. Baseline characteristics are shown in Appendix Supplemental Table 1. The mean patient age was 64.9 years; 56% were female; 14 (6.3%) were African American, 3 (1.4%) were Asian American, 1 (0.5%) was “other” race, and the remainder were White. Eight (3.6%) were of Hispanic ethnicity.

The body mass index (BMI) was slightly higher in the S2AI+TTI group; no other factors differed across the randomized groups. The 2-year rate of follow-up was 84%. Radiographic analysis was based on 174 CT scans (78% of all treated subjects), 171 full-spine radiographs (77%), and imaging prior to month 24 showing instrumentation failures.

Pelvic fixation failures meeting the primary radiographic end point definition were observed in 12 (11.0%) of the 109 patients assigned to S2AI+TTI versus 23 (20.4%) of the 113 assigned to S2AI alone (p = 0.0415). Because high BMI may be a risk factor for pelvic fixation failure^30^, logistic regression analyses controlling for BMI were performed. The univariate and bivariate odds ratios for the primary radiographic end point were 0.48 and 0.51, suggesting that the uneven distribution of BMI had little impact on the reduced likelihood of pelvic fixation failure in the S2AI+TTI group. Components of the primary radiographic end point are shown in Table 1. Except for rod screw dissociation and rod fracture distal to S1, all occurrence rates were lower in the S2AI+TTI group, but the differences were not significant. Rod fracture distal to S1 occurred in 1 subject in the S2AI+TTI group and none in the S2AI-alone group.

**Table 1:** Primary Radiographic End Point and Its Components

	Treatment Assignment		P Value
	S2AI+TTI (N = 109)	S2AI Alone (N = 113)	
Primary composite radiographic end point	12 (11.0%)	23 (20.4%)	0.0415
S2AI screw fracture	5 (4.6%)	8 (7.1%)	0.6323
Fracture along shaft	1 (0.9%)	2 (1.8%)	0.5136
Fracture at neck	0 (0.0%)	4 (3.5%)	0.0654
Rod-screw dissociation*	4 (3.7%)	3 (2.7%)	0.7918
S2AI lucency	8 (7.3%)	15 (13.3%)	0.1089
Rod fracture distal to S1	1 (0.9%)	0 (0.0%)	1
Lucency of S1 screw†	6 (5.5%)	7 (6.2%)	0.5271

* Includes set plug dissociation.

† Used in the modified primary radiographic end point calculation.

A prespecified modified primary end point analysis also included S1 screw fracture, lucency, or pullout (which occurred in 6 cases [5.5%] in the S2AI+TTI group versus 7 [6.2%] in the S2AI-alone group). The modified end point occurred in 13 cases (11.9%) versus 24 (21.2%), respectively (p = 0.0458).

### Secondary Radiographic End Points

Lucency in the sacral ala was lower in the S2AI+TTI group than in the S2AI-alone group (p = 0.03 [left] and p = 0.04 [right]) (Table 2). The number of regions along the S2AI screws that exhibited loosening was also lower in the S2AI+TTI group, but the difference was not significant.

**Table 2:** S2AI Loosening Grade by Location, Side, and Treatment

Location	Grade*	Treatment		P Value†
		S2AI+TTI (N = 88)	S2AI Alone (N = 87)	
Ilium, left				0.1665
	0	82 (93%)	78 (90%)	
	1	1 (1%)	0 (0%)	
	2a	0 (0%)	1 (1%)	
	2b	5 (6%)	8 (9%)	
Ilium, right				0.3332
	0	82 (93%)	78 (90%)	
	1	0 (0%)	2 (2%)	
	2a	1 (1%)	2 (2%)	
	2b	5 (6%)	5 (6%)	
Sacrum, left				0.0342
	0	84 (95%)	76 (87%)	
	1	0 (0%)	1 (1%)	
	2b	4 (5%)	10 (11%)	
Sacrum, right				0.0427
	0	83 (94%)	75 (86%)	
	1	0 (0%)	1 (1%)	
	2b	5 (6%)	11 (13%)	
No. of regions along screw				0.0603
	0	80 (91%)	72 (83%)	
	1	1 (1%)	3 (3%)	
	2	2 (2%)	4 (5%)	
	3	4 (5%)	2 (2%)	
	4	1 (1%)	6 (7%)	

* Grading per modified Shimizu-Lenke system^27^.

† Cochran-Armitage trend test.

The TTI were fully seated (i.e., at least 20 mm) into the sacrum in 78% of cases (Fig. 4; see also Appendix Supplemental Table 2). Adequate iliac engagement of the TTI was seen in 49 cases (83%) when navigation was used and 20 (69%) when fluoroscopy was used (p = 0.1698). Bone adjacent to the TTI was seen in the ilium in all cases and in the sacrum in all but 1 case (in which the TTI were placed within the SIJ but not in the sacrum). Bridging bone across the SIJ was seen in 23 (26%) of 88 S2AI+TTI cases and 16 (18.6%) of 86 S2AI-alone cases (p = 0.1454). Adverse bone reactions (primarily lucency) around TTI occurred in 12 (15%) of the cases in the S2AI+TTI group but did not prompt TTI removal.

**Fig. 4:**
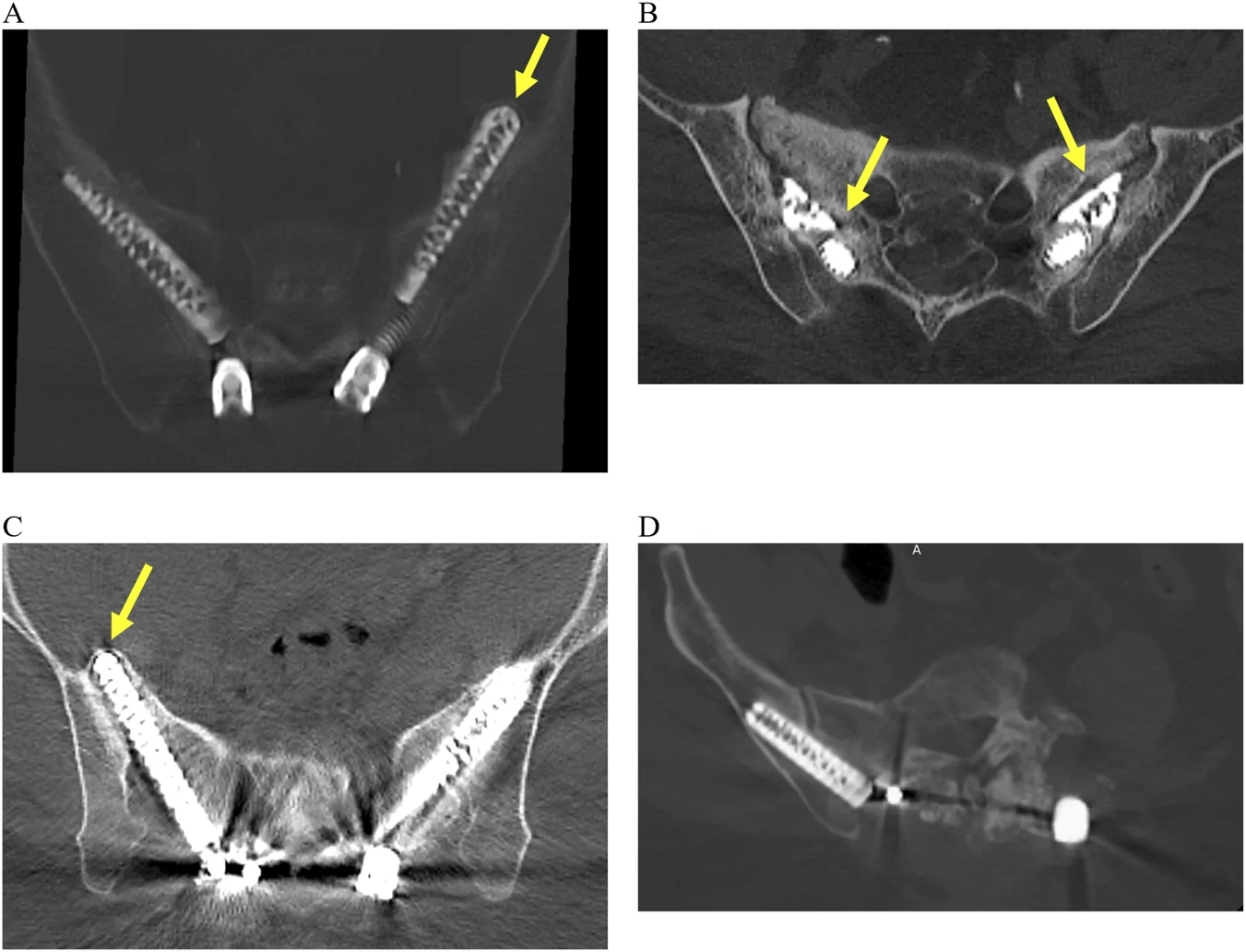
**Fig. 4-A:** The distal end of the TTI is outside of the ilium (yellow arrow). **Fig. 4-B:** Lucency is visible adjacent to the TTI (yellow arrows). **Fig. 4-C:** Sclerosis around the distal end of the TTI (yellow arrow). **Fig. 4-D:** TTI are placed within the SIJ and into the ilium but not seated well within the sacrum.

Distal rod fractures (at L3 or below) occurred in 10 (9.2%) of 109 cases in the S2AI+TTI group versus 6 (5.3%) of 113 in the S2AI-alone group (p = 0.197).

Baseline and 24-month sagittal and coronal alignment parameters were similar across groups, and changes at 24 months also showed no differences (see Appendix Supplemental Tables 3 and 4).

## Discussion

In our randomized trial, placement of TTI suprajacent to S2AI screws in patients undergoing multilevel spinal fusion yielded a lower rate of pelvic fixation failure (11.0% versus 20.4%, p = 0.0415). All components of the primary radiographic end point occurred at a lower rate in the S2AI+TTI group, except for rod-screw dissociation and rod fractures distal to S1. These findings support the study’s primary hypothesis that a second point of fixation across the SIJ protects the S2AI screw via a resulting reduction in SIJ motion, as suggested by preclinical^18-20,31^ and preliminary clinical^21,32^ studies. S2AI lucency was reduced in the S2AI+TTI group due to a reduced lucency rate within the sacrum. We did not observe any effect of TTI placement on the S1 screw lucency rate (5.5% for S2AI+TTI versus 6.2% for S2AI alone), which is inconsistent with prior preclinical studies of this construct^23,31^.

Although not significant, rod fractures from L3 to S1 were more common in the S2AI+TTI group (9.2%) than in the S2AI-alone group (5.3%). This suggests that the S2AI+TTI construct may increase proximal rod loading. Multirod constructs, anterior column load-sharing, optimized sagittal alignment, and enhanced bone healing (e.g., with biologics) may prevent rod fractures.

TTI performed well in our study, with no evidence of breakage or migration. Adverse bone reactions (15%, mostly lucency) were not associated with clinical sequelae or revision surgery. Adequate engagement of TTI into the ilium was more common when navigation systems were used, but the difference was not significant. Suprajacent TTI placement did not impact 24-month sagittal and coronal alignment parameters. We observed no effect of TTI on the change in pelvic incidence following ASD surgery.

Our study has important implications. We previously showed that placement of TTI suprajacent to S2AI screws was feasible, with a high rate of successful placement (106 of 109, 97%)^38^. The current study showed that this technique was associated with a reduced rate of adverse pelvic radiographic outcomes, which could reduce the overall high rate of pelvic fixation failures that is recognized. As previously noted, our companion study showed a reduced rate of postoperative new-onset SIJ pain^26^. Our current study suggests that use of 2 points of fixation across the SIJ during multilevel spinal fusion may have substantial benefits.

Our study used TTI, the only implant indicated at the study start for concomitant SIJ fusion. Since then, alternative 3D-printed porous devices for this indication have become available, including options allowing connection to posterior rods (iFuse Bedrock Granite; SI-BONE) or not allowing that (iFuse TORQ; SI-BONE)^33,34^. These porous-screw fusion devices integrate within bone and show higher removal torque^35^. The post-market surveillance experience with the former showed a low rate of failure^36^.

Although our study assigned treatment at random, BMI was slightly higher in the S2AI+TTI group, and BMI is a risk factor for pelvic fixation failure^30^. However, controlling for BMI in the analysis showed little impact on the study’s overall result. Further studies are needed to assess whether particular patient subgroups (e.g., with high or low pelvic incidence, or with a preoperative SIJ vacuum phenomenon^37^) and/or particular planned surgical approaches (e.g., large-magnitude sagittal contour corrections) benefit most from the technique that we studied.

Strengths of our study include its multicenter randomized approach, structured follow-up with high availability of CT scans, independent radiographic assessment, and use of objective outcomes. The study power was adequate to detect a 50% relative decrease in the rate of the primary end point (which is approximately what we observed).

Our study also had several weaknesses. The target patient population was highly variable in terms of underlying spinal deformity, resulting in marked variation in spinal fusion techniques and instrumentation, which could tend to blur any effect of the study’s intervention. Although the study was adequately powered for the primary end point, it was not powered for the individual end point components. Nevertheless, it was notable that the rates of most individual components were lower in the S2AI+TTI group. Potential study confounders, such as bone mineral density as well as pre- and postoperative use of biologics to enhance fusion, were not collected as part of this study. However, given the randomized controlled design, we expect these factors to be equally distributed between groups, thereby minimizing the potential for bias in the comparison. Finally, only 2-year follow-up was planned. Additional fixation failures could occur with further follow-up.

### Conclusions

In this multicenter randomized trial, placement of TTI suprajacent to S2AI screws in patients undergoing ASD surgery decreased the rate of pelvic fixation failure.

## Appendix

Supporting material provided by the authors is posted with the online version of this article as a data supplement at jbjs.org (http://links.lww.com/JBJS/J373).

## Data Availability

A **data-sharing statement** is provided with the online version of the article (http://links.lww.com/JBJS/J374).
